# Coronary artery bypass grafting is associated with immunoparalysis of monocytes and dendritic cells

**DOI:** 10.1111/jcmm.15154

**Published:** 2020-03-17

**Authors:** Alexis J. Perros, Arlanna Esguerra‐Lallen, Kelly Rooks, Fenny Chong, Sanne Engkilde‐Pedersen, Helen M. Faddy, Elise Hewlett, Rishendran Naidoo, John‐Paul Tung, John F. Fraser, Peter Tesar, Marc Ziegenfuss, Susan Smith, Donalee O’Brien, Robert L. Flower, Melinda M. Dean

**Affiliations:** ^1^ Research and Development Australian Red Cross Lifeblood Brisbane QLD Australia; ^2^ School of Medicine University of Queensland Brisbane QLD Australia; ^3^ Critical Care Research Group (CCRG) The Prince Charles Hospital Brisbane QLD Australia; ^4^ Adult Intensive Care Services The Prince Charles Hospital Brisbane QLD Australia; ^5^ Faculty of Health Queensland University of Technology Brisbane QLD Australia; ^6^ School of Health and Sport Sciences University of the Sunshine Coast Petrie QLD Australia; ^7^ Cardiothoracic Surgery Program The Prince Charles Hospital Brisbane QLD Australia

**Keywords:** cardiac surgery, coronary artery bypass, Dendritic cells, immune modulation, immunoparalysis, monocytes

## Abstract

Coronary artery bypass grafting (CABG) triggers a systemic inflammatory response that may contribute to adverse outcomes. Dendritic cells (DC) and monocytes are immunoregulatory cells potentially affected by CABG, contributing to an altered immune state. This study investigated changes in DC and monocyte responses in CABG patients at 5 time‐points: admission, peri‐operative, ICU, day 3 and day 5. Whole blood from 49 CABG patients was used in an *ex vivo* whole blood culture model to prospectively assess DC and monocyte responses. Lipopolysaccharide (LPS) was added in parallel to model responses to an infectious complication. Co‐stimulatory and adhesion molecule expression and intracellular mediator production was measured by flow cytometry. CABG modulated monocyte and DC responses. In addition, DC and monocytes were immunoparalysed, evidenced by failure of co‐stimulatory and adhesion molecules (eg HLA‐DR), and intracellular mediators (eg IL‐6) to respond to LPS stimulation. DC and monocyte modulation was associated with prolonged ICU length of stay and post‐operative atrial fibrillation. DC and monocyte cytokine production did not recover by day 5 post‐surgery. This study provides evidence that CABG modulates DC and monocyte responses. Using an *ex vivo* model to assess immune competency of CABG patients may help identify biomarkers to predict adverse outcomes.

## 1 INTRODUCTION

Coronary artery disease is the leading cause of death globally^1^ and can be treated by coronary artery bypass grafting (CABG). Exposure of patient blood to the bypass circuitry and re‐perfusion following CABG is associated with activation of leucocytes, complement and cytokine secretion.^2^ Activation of the patient’s inflammatory response following CABG has been suggested to contribute to, and be a potential indicator of adverse outcomes including infection, increased ICU length of stay (LOS), atrial fibrillation (AF), myocardial infarction, respiratory distress, multiple organ dysfunction (MOD), acute kidney injury and mortality.^3^, ^4^, ^5^

CABG can trigger the systemic inflammatory response syndrome (SIRS) characterized by leucocytosis, capillary leakage, MOD and an imbalance of pro‐inflammatory cytokine production (eg IL‐6, IL‐8).^6^, ^7^ SIRS is partially counteracted by the compensatory anti‐inflammatory response syndrome (CARS) characterized by the release of anti‐inflammatory cytokines (eg IL‐4, IL‐10) and has been reported to occur in up to 40% of CABG patients.^8^ An imbalance in immune homeostasis due to SIRS and/or CARS may increase the risk of CABG patients developing post‐operative infection, particularly when CARS dominates over SIRS.^9^ An imbalance in immune homeostasis can also lead to cell immunoparalysis, which renders the immune system unresponsive to a secondary insult (eg inflammation or infection).^10^, ^11^ Immunoparalysis or dysfunction of cells involved in the immune response could have detrimental implications for the patient, therefore restoring immune homeostasis is vital to prevent adverse patient outcomes.^12^, ^13^ In addition, preventative measures could be employed to manage the effects of a dysfunctional immune response. Changes to the patient immune profile following CABG can be divided into an early‐phase and late‐phase.^14^ Direct contact of patient blood with non‐endothelial surfaces associated with bypass circuitry triggers an early‐phase response activating multiple cellular pathways (eg coagulation, fibrinolysis, complement) and cell types (eg lymphocytes, monocytes, neutrophils).^15^ A late‐phase response is then triggered by neutrophil‐endothelial cell‐mediated heart/lung ischaemia and re‐perfusion injury.^14^, ^15^ Ischaemia damages endothelial cells resulting in neutrophil activation and the release of pro‐inflammatory mediators.^14^, ^15^

Pro‐inflammatory mediator release may also modulate immunoregulatory cells such as dendritic cells (DC) and monocytes. DC are antigen‐presenting cells that play a central role at the interface of the innate and adaptive immune response. In human peripheral blood, DC comprise < 1% of the leucocyte population and release cytokines and chemokines required for antigen presentation and immune regulation.^16^, ^17^, ^18^ Monocytes also link the innate and adaptive immune response and mediate antimicrobial host defence and removal of apoptotic cell debris.^19^, ^20^ Monocytes comprise 2%–12% of the leucocyte population and are a reservoir for myeloid precursors macrophages and DC.^19^ Understanding the role DC and monocytes play in CABG‐associated immunomodulation, and whether dysfunction in these cells contributes to adverse patient outcomes is limited. Therefore, we performed a prospective longitudinal assessment of the DC and monocyte phenotype in CABG patients at five time‐points: admission, peri‐operative, ICU, day 3 (D3) and day 5 (D5).

## 2 METHODS

### 2.1 Study approval and patient recruitment

Ethics approval (HREC/14/QPCH/117) was obtained from the Human Research Ethics Committee (HREC) at The Prince Charles Hospital (TPCH) which acts in accordance with the Declaration of Helsinki. Patients who were scheduled for CABG and met the inclusion criteria (Table 1) were enrolled with written informed consent. Whole blood (10 mL) was collected into an ethylenediaminetetraacetic acid (EDTA)‐anticoagulated phlebotomy tube (Becton Dickinson (BD)) at five time‐points—1) admission (just prior to anaesthesia but before surgery commencement), 2) peri‐operative (after chest closure), 3) ICU (24 h post), 4) D3 post‐operative and 5) D5 post‐operative.

**Table 1:** Inclusion criteria for study enrolment

Inclusion Criteria
Participant is scheduled for CABG
Participant has not received a transfusion of packed red blood cells or platelet concentrates in the past 5 days
Participant is not enrolled in any other research studies or clinical trials
Participant has not been diagnosed with human immunodeficiency virus, hepatitis c virus or hepatitis b virus infection
Participant is over the age of 18
English is the participant’s primary language
Participant is not pregnant
Participant is not of Aboriginal or Torres Strait Islander descent
Participant is not highly dependent on medical care
Participant does not have a cognitive impairment, intellectual disability or a mental illness
Participant is not involved in illegal activity

### 2.2 Anaesthesia and cardiopulmonary bypass

Anaesthesia was induced with a balanced anaesthesia protocol of propofol (0.5–1.5 mg/kg), fentanyl and rocuronium (0.6 mg/kg). Maintenance was achieved with a propofol infusion and an inhalational agent—sevoflurane. The bypass circuit was primed with colloid and crystalloid, with packed red blood cells (PRBC) being reserved for patients with low pre‐operative haemoglobin. Additives to the circuit included 2500 IU heparin, 20 mL of 8.4% sodium bicarbonate and 10 mL calcium chloride. The initial heparin dose was 300 IU/kg as a bolus maintaining the activated clotting time (ACT) > 400 s prior to aortic cannulation. The circuit included a hollow fibre membrane oxygenator, non‐occlusive roller pumps and a heat exchanger. Flows were calculated on a cardiac index of 2.4 L min^‐1^ m^‐2^ with perfusion pressures maintained at 50–70 mm Hg. Core temperatures were reduced to maintain moderate hypothermia (32–34°C), monitored by nasopharyngeal or bladder temperature probes. Myocardial preservation was achieved with St Thomas I crystalloid solution 30 mL/kg and maintained with blood cardioplegia—4:1 ratio. Heparin was reversed at the end of the procedure with protamine (1 mg for every 100 IU of heparin used).

### 2.3 Assessment of haematological parameters

Routine haematological parameters were determined by electronic impedance and absorption spectrophotometry (Abbott Cell‐Dyn Emerald Haematology Analyser). Reference ranges used were obtained from QML Pathology^21^ (Australia).

### 2.4 Ex vivo whole blood inflammatory response culture model

DC and monocyte specific responses (surface co‐stimulatory and adhesion molecule and intracellular cytokine production) were assessed using an established whole blood assay model.^22^ Patient blood and RPMI 1640 media (containing 2 mmol L^‐1^ L‐glutamine; Gibco by Life Technologies) were cultured 1:1 (1.5 mL total volume) in 2 wells of a 24‐well plate (Costar, Corning Life Sciences) for a total of 6 hrs (37°C 5% CO_2_). Golgi plug (containing brefeldin‐A; 1 μg/mL; BD Biosciences) was added after 1‐h incubation to facilitate detection of cytokines produced within the cell via flow cytometry. Duplicate plates were run in parallel with the addition of lipopolysaccharide (LPS; TLR4 specific *Escherichia coli* 055:B5; 1 μg/mL; Sigma) to model an infectious complication and investigate immunoparalysis.

### 2.5 Assessment of co‐stimulatory and adhesion molecules

For wells that did not receive Golgi plug, cells were harvested, centrifuged (1000 × g, 2 min) and surface‐stained with fluorescently labelled monoclonal antibodies to identify DC and monocyte populations (room temperature (RT), 15 min, dark; FITC Lineage Cocktail 2 [containing: CD3 (clone SK7), CD14 (clone MΦP9), CD19 (clone SJ25C1), CD20 (clone L27), CD56 (clone NCAM16.2)], CD45 PerCP (clone 2D1), CD11c APC (clone S‐HCL‐3), CD3 APC‐H7 (clone SK7), HLA‐DR V450 (clone L243)and CD14 V500 (clone M5E2); all BD Biosciences), followed by erythrocyte lysis (1 × FACS Lyse, BD Biosciences; RT, 10 min, dark). Leucocytes were then divided and stained with PE‐conjugated co‐stimulatory and adhesion molecules CD9 (clone M‐L13), CD38 (clone HIT2), CD40 (clone 5C3), CD80 (clone L307.4), CD83 (clone HB15e) or CD86 (clone FUN‐1); (RT, 30 min, dark; all BD Biosciences), washed (3% heat‐inactivated foetal calf serum (FCS)/phosphate‐buffered saline (PBS; both from Gibco by Life Technologies)) and resuspended in 1 × Stabilizing Fixative (BD Biosciences) for flow cytometric analysis.

### 2.6 Assessment of cytokine production

For wells that received Golgi plug, cells were harvested, centrifuged (1000 x g, 2 min) and stained with fluorescently labelled monoclonal antibodies to identify DC and monocyte populations as above (see ‘Assessment of co‐stimulatory and adhesion molecules’). Erythrocytes were then lysed (1 × FACS Lyse, RT, 10 min, dark), and leucocytes were permeabilized (1 × FACS Perm, RT, 10 min, dark) and then divided and stained with either PE‐conjugated anti‐mouse IgG1 (isotype control; clone MOPC‐21), anti‐rat IgG2a (isotype control; clone R35‐95), anti‐human IL‐6 (clone MQ2‐6A3), IL‐8 (clone G265‐8), IL‐10 (clone JES3‐19F1), IL‐12 (clone C11.5), IL‐1α (clone 364‐3B3‐14), TNF‐α (clone Mab11), MIP‐1α (clone 11A3), MIP‐1β (clone D21‐1351), MCP‐1 (clone 5D3‐F7) or IP‐10 (clone 6D4/D6/G2) (RT, 30 min, dark; all BD Biosciences). Cells were then washed in 3% FCS/PBS and resuspended in 1 × Stabilizing Fixative for flow cytometric analysis. Representative histograms of intracellular cytokine staining across each time‐point are provided in Figure S1.

### 2.7 Flow cytometry

A 3‐laser BD FACSCanto™ II flow cytometer with FACS Diva software (both BD Biosciences) was used for all flow cytometry and associated analyses. DC were gated as Lineage^‐^, HLA‐DR^+^, CD11c^+^ and monocytes were gated as CD14^+^ (with clone M5E2). For all conditions, an average of 1000 monocyte events and 500 DC events were collected.

### 2.8 Clinical data

Patient clinical data were obtained from prospectively collected records in the Queensland Cardiac Outcomes Registry (QCOR), the ICU Clinical Information System and Queensland Health electronic data repositories and medical records as required. Definitions are aligned with the Australian and New Zealand Society of Cardiothoracic Surgery Database (https://anzscts.org/database/), to which QCOR contributes to and is audited by. Clinical characteristics collected include demographics, relevant comorbidities, peri‐operative parameters including ICU LOS, ventilation time, post‐operative morbidity including new AF, sternal infection and other non‐cardiac complications (eg pneumonia).

### 2.9 Statistical analysis

A repeated measures one‐way ANOVA with Dunnett’s post‐test (with admission sample as the comparator) was used for analyses of haematological parameters, co‐stimulatory and adhesion molecules and cytokine production at each time‐point (*P *< 0.050; GraphPad Prism 7, GraphPad software). Monocytes and DC express HLA‐DR; therefore, we used the median fluorescent intensity (MFI) to assess changes in monocyte and DC HLA‐DR expression. The percentage of positive (%POS) cells was used to assess changes to the monocyte and DC immune profile. MFI was used to assess changes to the monocyte and DC immune profile when co‐stimulatory and adhesion marker expression or cytokine production was ≥ 90% at admission. Using the admission sample as the comparator, a ratio was calculated at each time‐point to determine changes from baseline. The log_2_ of this ratio was then calculated to equalize data around zero. In this way, +1 indicates a 2‐fold increase and −1 indicates a 2‐fold decrease. Differences in inflammatory responses were also investigated for association with clinical outcomes post‐operative AF and prolonged ICU LOS (long > 24 h; Spearman’s correlation, *P* < 0.050; GraphPad Prism 7).

## 3 RESULTS

### 3.1 Patient characteristics and basic haematological parameters

The cohort of 49 patients was predominantly male (90%), and the average age at surgery was 68 years (Table 2). The average ICU LOS for the patient cohort was 40 h, and of the 49 patients, 17 developed post‐operative AF (Table 2). As expected, CABG resulted in significant modulation of patient’s basic haematological parameters which include an increased white blood cell count over the post‐operative period and a reduced red blood cell count and haemoglobin levels particularly during CABG (Table 3).

**Table 2:** Patient demographic and clinical summary

Patient characteristics summary (n = 49)			
	n	Mean	SD
Patient characteristics			
Age (years)	49	68	±9
Sex			
Male	44		
Female	5		
History of smoking	38		
History of diabetes	20		
Clinical characteristics			
Total LOS ICU (Hours)	49	40	± 46
Ventilation Time (Hours)	49	9	± 5
Development of Atrial Fibrillation	17		
Development of Sternal Infection Development	4		
Other Complications (eg respiratory failure, tamponade)	7		

**Table 3:** Haematological parameters

Patient haematological characteristics (n = 49)						
Haematological characteristics	Admission	Operating theatre	ICU	Day 3	Day 5	Reference range^21^
White blood cell count (x10^9^/L)	6.2 ± 2.2	10.0 ± 3.5^****^	12.0 ± 4.3^****^	12.6 ± 4.3^****^	8.7 ± 2.3^****^	4.0–11.0
Red blood cell count (x10^12^/L)	4.1 ± 0.6	3.3 ± 0.6^****^	3.6 ± 0.8^***^	3.7 ± 1.0	3.5 ± 0.9^**^	3.6–6.0
Platelet count (x10^9^/L)	216 ± 64	162 ± 44^****^	196 ± 64	183 ± 50^*^	252 ± 79	150–450
Haemoglobin (g/L)	131 ± 18	107 ± 20^****^	116 ± 27^**^	119 ± 31	112 ± 26^**^	115–180
Haematocrit (L/L)	0.37 ± 0.05	0.31 ± 0.06^***^	0.33 ± 0.07^**^	0.34 ± 0.08	0.32 ± 0.08^**^	0.33–0.52
Mean cell volume (fL)	90 ± 5	94 ± 5^****^	91 ± 5^****^	92 ± 5^****^	91 ± 5	80–98
Mean cell haemoglobin (pg)	32.1 ± 2.2	33.6 ± 10.3	32.2 ± 2.2	32.4 ± 2.2	32.0 ± 2.3	27–35
Mean cell haemoglobin concentration (g/L)	355 ± 15	343 ± 15	352 ± 15	354 ± 15	352 ± 17	310–365

Note

Repeated measures one‐way ANOVA with Dunnett’s post‐test indicated by: ^*^ *P* < 0.05;^**^ *P* < 0.01;^***^ *P* < 0.001;^****^ *P* < 0.0001.

Data represent mean ± SD.

### 3.2 Expression of monocyte and DC co‐stimulatory and adhesion molecules were modulated following CABG

**Figure 1:**
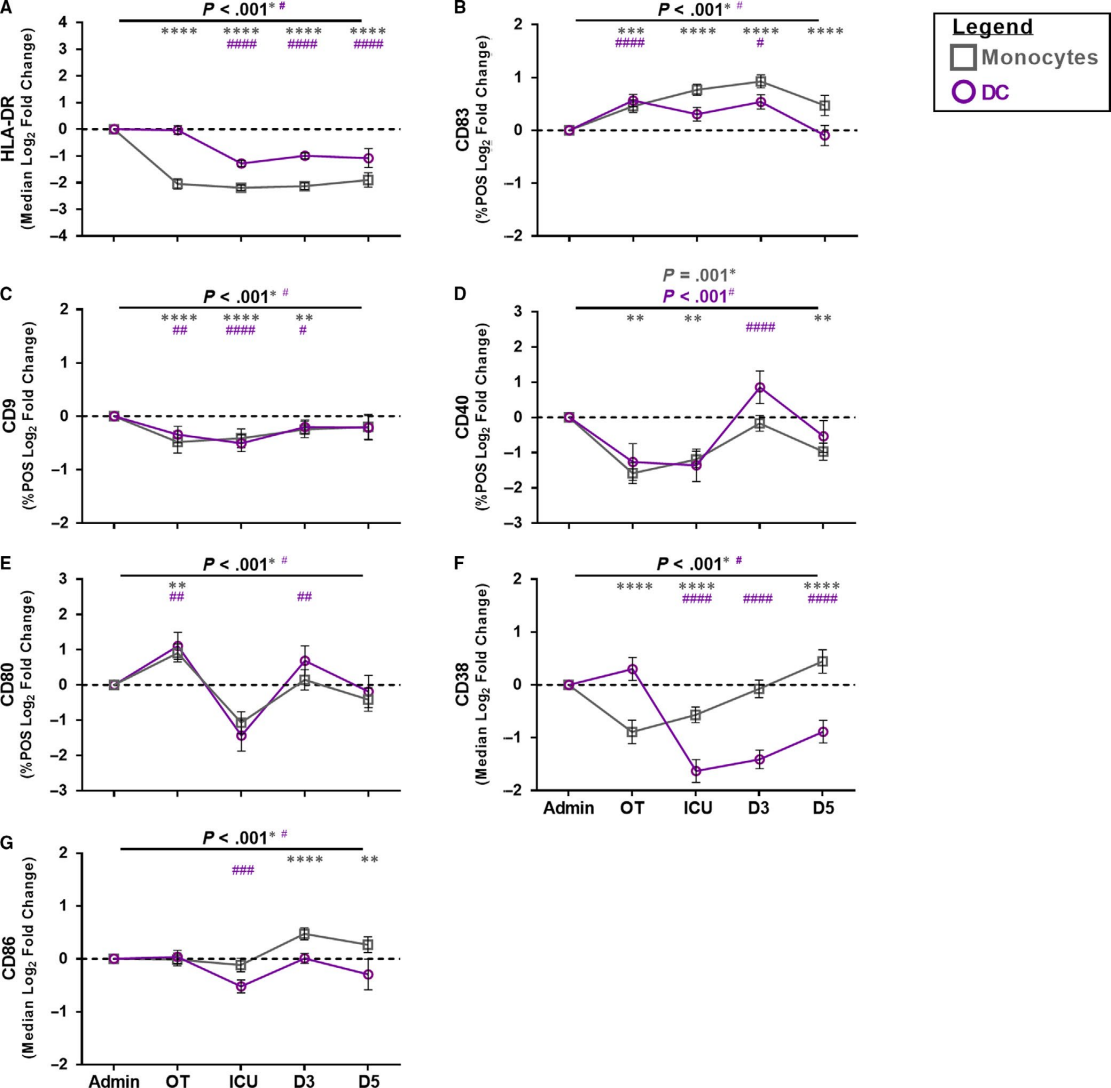
Assessment of monocyte and DC‐specific activation profile following CABG. Open grey squares indicate monocytes and open purple circles indicate DC. Y‐axis indicates the log_2_ fold changed data. X‐axis indicates sample collection time‐point (Admission (Admin)) and peri‐operative (OT), ICU, D3, D5). Data from 49 CABG patients. Symbols and bars at each time‐point represent mean ± SEM. As monocyte and DC expression of CD38 and CD86 approached 100%, changes in the expression were assessed using MFI. ANOVA indicated by horizontal bar with *P* value above. An asterisk indicates *P* value for monocytes and a hash indicates *P* value for DC. Dunnett’s post‐test (admission *vs.* sample time‐point) is indicated at specific time‐points by an asterisk for monocytes or hash for DC as follows: *^(or #)^ *P* < 0.050, **^(or ##)^ *P* < 0.010, ***^(or ###)^ *P* < 0.001, ****^(or ####)^ *P* < 0.0001

Reduced monocyte HLA‐DR expression has been reported to be an indicator of patient immunosuppression.^23^, ^24^ In this study, monocyte HLA‐DR expression was suppressed during CABG, and this suppression persisted throughout the post‐operative period (Figure 1A). We further investigated the monocyte activation status using a panel of co‐stimulatory and adhesion molecules. Monocyte expression of CD83 was increased during CABG and remained above admission levels through to D5 post‐surgery (Figure 1B). Monocyte expression of CD9 and CD40 was suppressed over the post‐operative period (Figure 1C–D). Expression of monocyte CD80 changed through the time‐course with an increase evident during surgery followed by a period of suppression post‐surgery (Figure 1E). As expression of CD38 and CD86 approached 100% on monocytes, we assessed changes in the expression of these molecules using the MFI. Monocyte expression of CD38 was suppressed over the post‐operative period until D5 where an increase was evident (Figure 1F). Meanwhile, monocyte CD86 expression increased at D3 and D5 post‐surgery (Figure 1G). By D5 post‐surgery, monocyte expression of CD9 and CD80 returned towards baseline levels, while monocyte expression of HLA‐DR, CD83, CD40, CD38 and CD86 did not.

DC are key immune mediators required to regulate and activate B cells, T cells and natural killer cells.^16^, ^17^, ^18^ Similar to monocyte HLA‐DR expression, DC HLA‐DR expression was suppressed during CABG, and this suppression persisted throughout the post‐operative period (Figure 1A). To date, the DC phenotype in patients who have had CABG has not been extensively characterized. DC expression of CD9 and CD38 was suppressed during and 24 h post‐CABG (Figure 1C, F). Of note, increased DC CD38 expression was evident during the CABG procedure before decreasing 24 h post‐surgery. DC expression of CD83, CD80 and CD40 was modulated during CABG (Figure 1B, D‐E). DC CD83 expression was induced during CABG and persisted to D3. Interestingly, significant elevation in CD80 during and D3 post‐surgery and CD40 expression at D3 was evident before a period of suppression. MFI was used to assess changes in expression of DC CD38 and CD86. Following CABG and over the post‐operative period, DC CD38 and CD86 expression was suppressed (Figure 1F‐G). While DC expression of CD83, CD9, CD40 and CD80 returned towards baseline levels by D5 post‐surgery, DC HLA‐DR and CD38 expression did not.

### 3.3 CABG suppressed monocyte and DC cytokine and chemokine production

**Figure 2:**
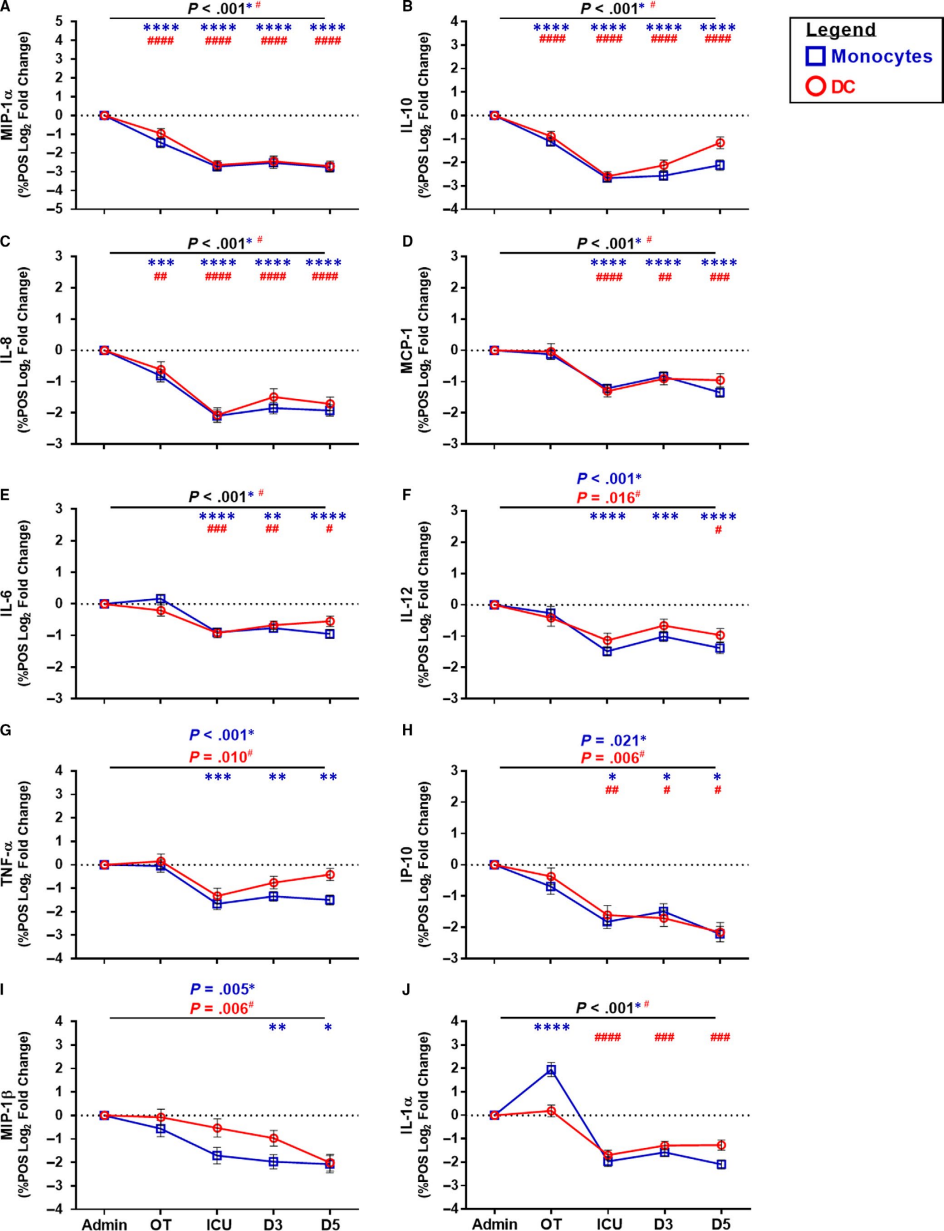
Assessment of the monocyte and DC‐specific immune profile following CABG. Open blue squares indicate monocytes and open red circles indicate DC. Y‐axis indicates the log_2_ fold change data. X‐axis indicates sample collection time‐point (Admission (Admin)) and peri‐operative (OT), ICU, D3, D5). Data from 49 CABG patients. ANOVA indicated by horizontal bar with *P* value above. An asterisk indicates *P* value for monocytes and a hash indicates *P* value for DC. Dunnett’s post‐test (admission *vs.* sample time‐point) is indicated at specific time‐points by an asterisk for monocytes or hash for DC as follows: *^(or #)^ *P* < 0.050, **^(or ##)^ *P* < 0.010, ***^(or ###)^ *P* < 0.001, ****^(or ####)^ *P* < 0.0001

Dysfunction of monocyte cytokine and chemokine signalling capacity can impact downstream processes associated with the patient immune response.^19^, ^20^ Monocyte production of MIP‐1α, IL‐10 and IL‐8 was significantly suppressed during CABG and throughout the post‐operative period (Figure 2A‐C). In addition, monocyte production of MCP‐1, IL‐6, IL‐12, TNF‐α, IP‐10 and MIP‐1β was also suppressed from the ICU period through to D5 post‐CABG (Figure 2D‐I). Of note, while monocyte IL‐1α production was increased during CABG, it was followed by a period of post‐operative suppression (Figure 2J). Monocyte cytokine and chemokine levels did not return to baseline levels by D5 post‐surgery.

DC cytokine and chemokine production is an essential secondary signal to trigger antigen presentation and induce T‐cell priming towards a pro‐inflammatory state.^25^ DC capacity to produce IL‐10, MIP‐1α and IL‐8 was significantly suppressed during surgery and remained below admission levels through to D5 post‐CABG (Figure 2A‐C). DC production of IL‐1α, MCP‐1, IL‐6, IP‐10, IL‐12, MIP‐1β and TNF‐α was also suppressed during CABG (Figure 2D‐J). DC capacity to produce cytokines and chemokines did not recover by D5 post‐surgery.

### 3.4 Monocyte and DC co‐stimulatory and adhesion molecule expression was immunoparalysed following CABG

**Figure 3:**
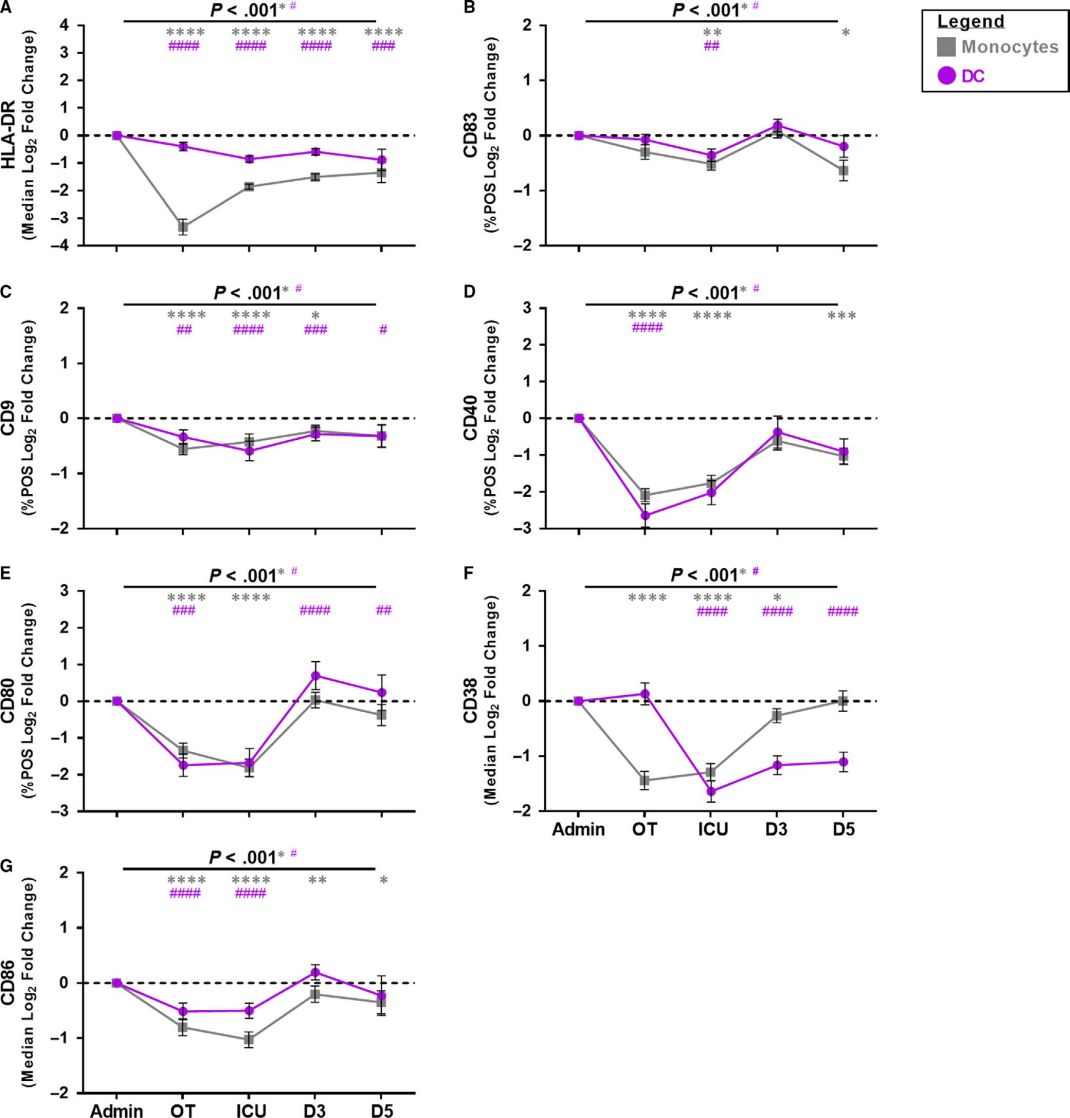
Assessment of monocyte and DC‐specific activation profile following CABG in a model of bacterial infection. Closed grey squares indicate monocytes and closed purple circles indicate DC. Y‐axis indicates the log_2_ fold changed data. X‐axis indicates sample collection time‐point (Admission (Admin)) and peri‐operative (OT), ICU, D3, D5). Data from 49 CABG patients. Symbols and bars at each time‐point represent mean ± SEM. As monocyte and DC expression of CD38 and CD86 approached 100%, changes in the expression were assessed using MFI. ANOVA indicated by horizontal bar with *P* value above. An asterisk indicates *P* value for monocytes and a hash indicates *P* value for DC. Dunnett’s post‐test (admission *vs.* sample time‐point) is indicated at specific time‐points by an asterisk for monocytes or hash for DC as follows: *^(or #)^ *P* < 0.050, **^(or ##)^ *P* < 0.010, ***^(or ###)^ *P* < 0.001, ****^(or ####)^ *P* < 0.0001

Immunoparalysis is a form of acquired immunodeficiency which renders the patient immune system unresponsive to secondary insult (eg LPS) as a result of an imbalance in immune homeostasis.^26^, ^27^ In this study, we used an *ex vivo* culture model of bacterial infection to assess patient monocyte and DC responses to LPS as the bacterial stimulus. Monocyte expression of HLA‐DR was down‐regulated and had a reduced capacity to respond to LPS during CABG and over the post‐operative period (Figure 3A). Monocyte expression of CD83, CD9, CD40 and CD80 was also suppressed and had a reduced capacity to respond to LPS during surgery and in the ICU period (Figure 3B‐E). As expression of CD38 and CD86 approached 100% on monocytes, we assessed changes in co‐stimulatory and adhesion molecule expression using the MFI. Monocyte CD38 and CD86 expression were reduced and had a deficiency in their capacity to respond to LPS during CABG and over the post‐operative period (Figure 3F‐G). Monocyte co‐stimulatory and adhesion molecules failed to respond to LPS (secondary stimuli) indicating immunoparalysis, which was evident throughout the post‐operative period. LPS‐stimulated monocyte co‐stimulatory and adhesion molecule expression of CD9, CD80 and CD38 returned near baseline levels post‐surgery, but monocyte HLA‐DR, CD83, CD40 and CD86 expression did not.

While immunoparalysis has been reported previously in mice spleen–derived DC and in a model of differentiated MoDC from sepsis patients following secondary insult,^11^, ^28^, ^29^, ^30^, ^31^ understanding of DC immunoparalysis is limited. In the model of bacterial infection, DC HLA‐DR expression was down‐regulated and had a reduced capacity to respond to LPS during CABG and throughout the entire post‐operative period (Figure 3A). Further investigation of the DC activation status revealed that patient DC were immunoparalysed to LPS stimulation. DC expression of CD9, CD83, CD80 and CD40 was suppressed and had a reduced capacity to respond to LPS during surgery and 24 h post‐CABG in ICU (Figure 3B‐E). Changes in DC CD38 and CD86 expression were assessed using the MFI. DC CD38 and CD86 expression was suppressed and had a deficiency in its capacity to respond to LPS during CABG, and over the post‐operative period (Figure 3F‐G). DC co‐stimulatory and adhesion molecules failed to respond to LPS (secondary stimuli) indicating immunoparalysis, evident throughout the post‐operative period. In the bacterial infection model, DC expression of CD83, CD40 and CD86 recovered by D5 to baseline levels. However, LPS‐stimulated DC expression of HLA‐DR, CD9, CD80 and CD38 failed to return to baseline levels by D5 post‐surgery, suggesting DC may have an impaired capacity for antigen presentation persistent beyond D5 post‐CABG.

### 3.5 Monocyte and DC cytokine production was immunoparalysed following CABG

**Figure 4:**
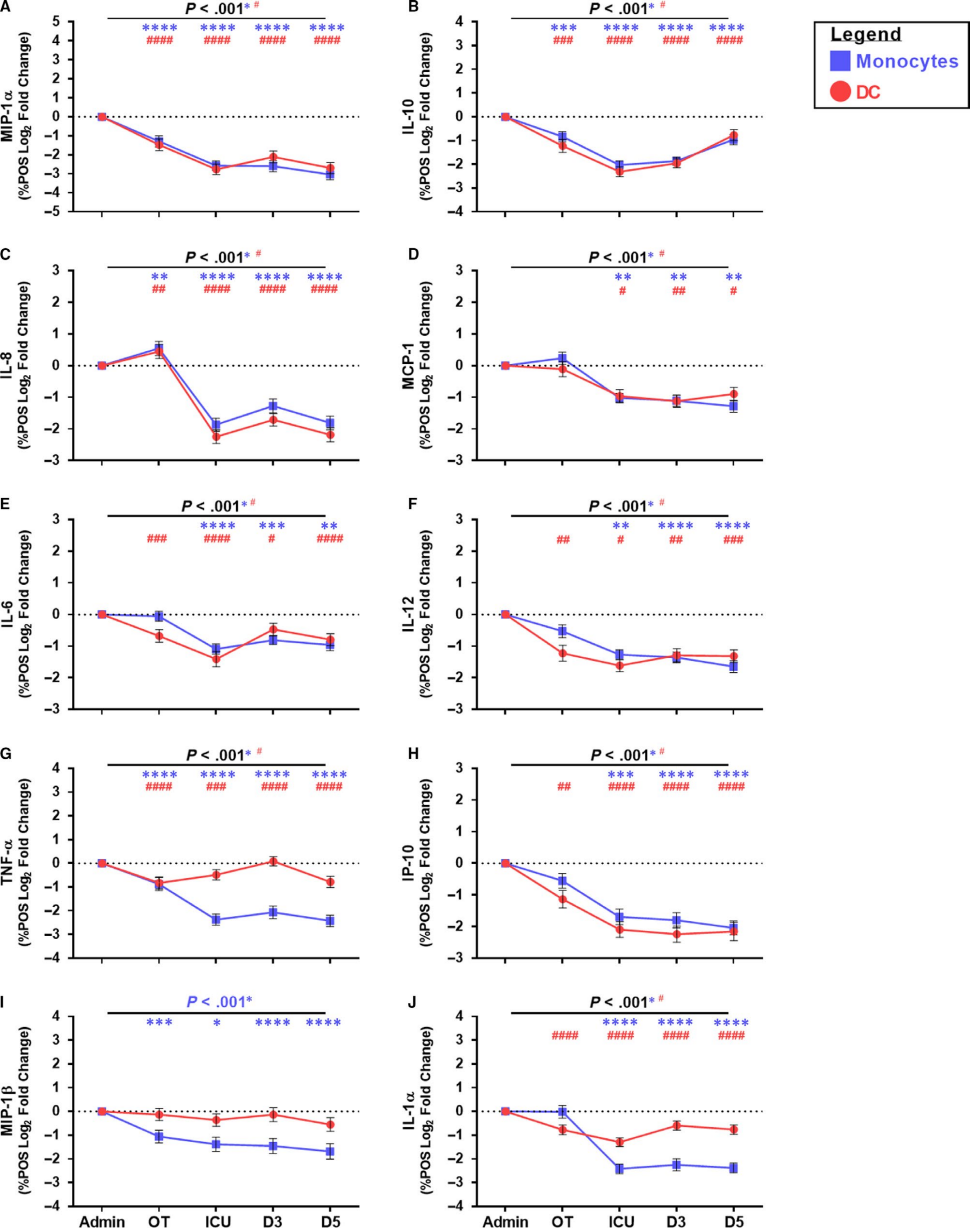
Assessment of the monocyte and DC cytokine production following CABG in a model of bacterial complications. Closed blue squares indicate monocytes and closed red circles indicate DC. Y‐axis indicates the log_2_ fold change data. X‐axis indicates sample collection time‐point (Admission (Admin)) and peri‐operative (OT), ICU, D3, D5). Data from 49 CABG patients. Symbols and bars at each time‐point represent mean ± SEM. ANOVA indicated by horizontal bar with *P* value above. An asterisk indicates *P* value for monocytes and a hash indicates *P* value for DC. Dunnett’s post‐test (admission *vs.* sample time‐point) is indicated at specific time‐points by an asterisk for monocytes or hash for DC as follows: *^(or #)^ *P* < 0.050, **^(or ##)^ *P* < 0.010, ***^(or ###)^ *P* < 0.001, ****^(or ####)^ *P* < 0.0001

Utilizing the *ex vivo* model of bacterial infection, we found patient monocytes and DC were immunoparalysed following CABG, evidenced by a deficiency of monocytes and DC to produce cytokines and chemokine in response to LPS stimulation. Monocyte production of MIP‐1α, IL‐10, IL‐8, TNF‐α and MIP‐1β was suppressed and had a reduced capacity to respond to LPS during surgery and over the entire post‐operative period (Figure 4A‐C, G, I). Of note, monocyte IL‐8 production increased during CABG, before a period of suppression (Figure 4C). In addition, monocyte production of MCP‐1, IL‐6, IL‐12, IP‐10 and IL‐1α was down‐regulated and had a reduced capacity to respond to LPS from 24 h post‐CABG (Figure 4D‐F, H, J). Monocytes failed to respond to LPS (secondary stimuli) indicating immunoparalysis. Monocyte immunoparalysis was evident throughout the post‐operative period and monocyte cytokine and chemokine production capacity did not return near to baseline levels by D5 post‐surgery.

DC production of IL‐10, MIP‐1α, IL‐8, IL‐1α, IL‐6, IP‐10, IL‐12 and TNF‐α was down‐regulated and had a reduced capacity to respond to LPS during CABG and over the post‐operative period (Figure 4A‐C, E‐H, J). DC MIP‐1β production was not significantly modulated in the bacterial infection model compared to admission. DC failed to respond to LPS (secondary stimuli) indicating immunoparalysis. DC immunoparalysis was evident by reduced cytokine production throughout the post‐operative period. LPS‐stimulated DC capacity to produce cytokines and chemokines did not recover by D5 post‐surgery.

### 3.6 Correlation of findings with clinical patient outcomes

Preliminary analyses of clinical outcomes ICU LOS and post‐operative AF and their association with *ex vivo* changes to the monocyte and DC phenotype for the 49 CABG patients were conducted. Of the 49 patients recruited in this study, 17 (34.6%) developed post‐operative AF. This is not significantly different to the entire TPCH incidence of post‐operative AF. Modulated DC expression of CD38 at D3 (*P* = 0.040; *R* = −0.294) and DC production of IL‐8 at D5 (*P* = 0.045; *R* = 0.288) was associated with the development of post‐operative AF in the absence of LPS stimulation. In an *ex vivo* model of bacterial complications, monocyte production of IL‐10 (*P* = 0.021; *R* = 0.330) was associated with post‐operative AF during the ICU period.

Patient ICU LOS was divided into either short or long based on site‐wide data from TPCH where long ICU LOS was considered to be > 24 h (*vs.* short ≤ 24 h). The mean ICU LOS of the CABG patient cohort was 40 h (± 46 h). Prolonged ICU LOS following CABG was associated with decreased expression of DC CD9 (*P* = 0.040; *R* = −0.295), increased DC CD83 expression (*P* = 0.026; *R* = −0.318) during surgery, decreased DC production of IL‐8 at admission (*P* = 0.009; *R* = −0.372), IL‐1α at admission (*P* = 0.010; *R* = −0.366), IL‐12 during surgery (*P* = 0.002; *R* = −0.425), MIP‐1α during surgery (*P* = 0.037; *R* = −0.299) and during the ICU period (*P* = 0.012; *R* = −0.358) and TNF‐α at D3 (*P* = 0.029; *R* = 0.312). Prolonged ICU LOS following surgery was also associated with decreased monocyte production of IL‐8 at admission (*P* = 0.020; *R* = −0.331), MCP‐1 at admission (*P* = 0.037; *R* = −0.298) and MIP‐1α during surgery (*P* = 0.033; *R* = −0.306). In an *ex vivo* whole blood culture model, with the addition of LPS in parallel to model a bacterial complication, decreased monocyte expression of CD38 at D3 (*P* = 0.039; *R* = −0.296), decreased monocyte production of IL‐8 at admission (*P* = 0.005; *R* = −0.396), decreased DC production of IL‐8 at admission (*P* = 0.001; *R* = −0.454), IL‐12 at admission (*P* = 0.039; *R* = −0.295), IL‐1α at admission (*P* = 0.035; *R* = −0.303), MIP‐1α during the ICU period (*P* = 0.026; *R* = −0.318), MCP‐1 during the ICU period (*P* = 0.030; *R* = −0.311) and DC expression of CD9 during surgery (*P* = 0.029; *R* = −0.312) were associated with prolonged ICU LOS. Post‐operative sternal wound infection was low, occurring in only 4 (8.2%) patients.

## 4 DISCUSSION

CABG remains as one of the most common cardiac surgery procedures worldwide.^32^ Our study provides a comprehensive assessment of monocyte and DC responses in CABG patients which were significantly modulated post‐surgery. Monocytes and DC play a central role mediating the immune response through cytokine release and co‐stimulatory molecule presentation. Deficiency in co‐stimulatory and adhesion molecule expression may impede cellular function and increase the risk of post‐operative infection. To date, understanding of immunomodulation of the DC and monocyte immune profile in CABG patients is limited.

We investigated the monocyte and DC co‐stimulatory and adhesion molecule expression profile in CABG patients and demonstrated that both DC and monocyte HLA‐DR, co‐stimulatory and adhesion molecule expression were suppressed following CABG and throughout the post‐operative period. Modulated co‐stimulatory and adhesion molecule function following CABG may impair host defence mechanisms including cell migration, antigen presentation to naïve T cells and T‐cell regulation, therefore increasing the risk of post‐operative infection. Down‐regulated monocyte HLA‐DR and CD86 expression, and up‐regulated monocyte CD40 and CD80 expression have also been reported in septic patients.^33^, ^34^ Reduced HLA‐DR and CD86 expression has been hypothesized to be in part due to reduced antigen presentation in these patients.^34^ However, the underlying mechanisms that drive immunomodulation of host defences in sepsis and surgical patients remain unclear. Infective complications in our cohort were relatively low, with the sternal wound infection rate 8.2%.

Monocyte and DC pro‐inflammatory and anti‐inflammatory cytokine signalling is important mediating the adaptive and innate immune response against pathogens and foreign bodies.^35^, ^36^, ^37^, ^38^ We found evidence of suppressed monocyte and DC cytokine and chemokine production in patients who underwent CABG. Dysfunctional cytokine and chemokine signalling processes may be associated with clinical complications post‐CABG and increase the risk of post‐operative infection due to impaired antimicrobial defences, and regulation and activation of T and B cells. This may translate into increased infective complications or those related to the pro‐inflammatory status associated with cardiopulmonary bypass (eg AF). In CABG patients, restoring immune homeostasis following modulated pro‐inflammatory cytokine production (eg IL‐6, IL‐8) and/or anti‐inflammatory cytokine production (eg CXCL16, IL‐10), as observed in our study, is key to reducing the risk of post‐operative complications including secondary infection;^13^, ^39^, ^40^ however, further investigation is required.

Persistent imbalance of immune homeostasis can result in a state of overwhelming immunosuppression and consequently immunoparalysis.^10^, ^11^, ^12^ Immunoparalysis is hypothesized to be a protective adaptation to an overwhelming pro‐inflammatory response rendering key cells of the immune response unresponsive to a secondary insult (eg LPS).^10^, ^11^ When cells become paralysed, their capacity to respond appropriately to additional stimulus is reduced^10^, ^11^ and is characterized by reduced monocyte HLA‐DR surface expression and TNF‐α production.^41^, ^42^ As a result, patients with immunoparalytic cells may have an increased risk of infectious complications following trauma or surgery. The effects of immunoparalysis have been reported in trauma,^43^ sepsis,^42^ cardiac surgery^26^, ^41^ and in the ICU setting.^27^

While down‐regulation of DC HLA‐DR expression in sepsis patients has been demonstrated,^24^ comprehensive understanding of DC immunoparalysis in CABG patients is limited. We demonstrated that using LPS to model a bacterial complication, changes to the DC and monocyte activation status was also evident. Monocytes and DC were immunoparalysed, evidenced by a failure to respond to a secondary insult (LPS). While most DC and monocyte activation markers returned towards pre‐operative levels by D5, monocyte HLA‐DR expression and DC expression of HLA‐DR, CD9 and CD38 failed to return to baseline by D5 post‐surgery in the model of bacterial infection, suggesting impaired antigen presentation and cell migration. DC CD9, CD38 and HLA‐DR expression help mediate T‐cell activation^44^ and immunoparalysis post‐surgery, evidenced by reduced expression on DC, could result in dysregulated antigen presentation and T‐cell activation reducing host defences against pathogens or foreign bodies.

Dysfunction to monocyte and DC cytokine production may result in an imbalance in immune homeostasis, which could also lead to immunoparalysis. Using LPS as the stimulant to model a bacterial infection, monocyte and DC cytokine production was immunoparalysed following CABG and over the entire post‐operative period. In contrast to the assessment of monocyte and DC co‐stimulatory and adhesion molecule expression, recovery of the monocyte and DC cytokine profile was not observed over the post‐operative period, suggesting persistent cytokine and chemokine signalling dysfunction that may affect antimicrobial defences, cellular recruitment and increased risk of post‐operative infection and prolonged ICU LOS. LPS‐induced immunoparalysis of key pro‐inflammatory cytokines (eg IL‐6, IL‐8, IL‐10, IL‐12, TNF‐α) released from monocytes has also been previously reported in patient plasma and supernatants collected from *in vitro* culture models investigating healthy donors,^45^ cardiac surgery patients^41^ and sepsis patients.^7^, ^30^, ^46^, ^47^ However, immunoparalysis of DC cytokine production is not well characterized and the underlying mechanisms which drive immunoparalysis remain unknown, warranting further investigation.

AF is reported as the most frequent post‐operative complication observed in the cardiac setting, with 30%–50% of patients undergoing cardiac surgery developing AF post‐operatively. This prolongs admission, may require anticoagulation if persistent and is associated with poorer patient outcomes.^48^ Prolonged ICU LOS can impede long‐term patient recovery and increase hospital expenses.^49^ This is multifactorial, being influenced by infection, ventilation time and the need for blood transfusion.^49^ Preliminary findings from our study suggest a modulated DC and monocyte phenotype is associated with prolonged ICU LOS and post‐operative AF. Early prediction of the risk for adverse outcomes may guide early patient intervention and management in order to minimize post‐operative complications, improving patient outcomes. Post‐operative AF is difficult to prevent, and while we do not suggest that these changes are solely responsible for it, it may provide avenues for further study in terms of prevention. While this study investigates the CABG patient immune profile, our findings warrant further investigation of predictive markers of adverse outcomes.

We provide evidence that CABG suppresses the monocyte and DC immune profile, and LPS exposure in the *ex vivo* model of bacterial infection results in immunoparalysis. This may, in turn, increase the risk of post‐operative complications such as infection.^50^ We found dysfunction of the DC and monocyte immune profile at admission, during surgery and throughout the post‐operative period was associated with prolonged ICU LOS and post‐operative AF. Our comprehensive assessment of the DC and monocyte immune profile using an *ex vivo* culture model could be used to assess the immune profile in other patient groups. Assessment of immune competency following CABG may enable early prediction of adverse outcomes such as prolonged ICU LOS and post‐operative AF, helping guide clinical decision‐making and patient management.

## CONFLICTS OF INTEREST

Dr. Perros reports a new investigator grant from The Prince Charles Hospital Foundation during the conduct of the study. All other authors confirm no conflicts of interest.

## AUTHOR CONTRIBUTIONS

MD conceptualized and designed the study. AP, KR, FC, EH and MD performed experimental work. AE and SE conducted patient recruitment. AE, SE, SS and RN collected in‐hospital data. JPT, RN, JF, PT, MZ, DO and RF contributed to study progression. AP, MD and HF analysed and interpreted the data. AP and MD prepared manuscript. All authors contributed to drafting of final manuscript.

## Supporting information

Fig S1

## Data Availability

The data that support the findings of this study are available on request from the corresponding author. The data are not publicly available due to privacy or ethical restrictions.
