# Supplementary material for: Coronary artery bypass grafting is associated with immunoparalysis of monocytes and dendritic cells
Source: J Cell Mol Med. 2020 Mar 17;24(8):4791–803. doi: 10.1111/jcmm.15154 (PMC7176880; doi:10.1111/jcmm.15154)
Supplement: Supplementary file 1 — Fig S1 [file JCMM-24-4791-s001.docx]

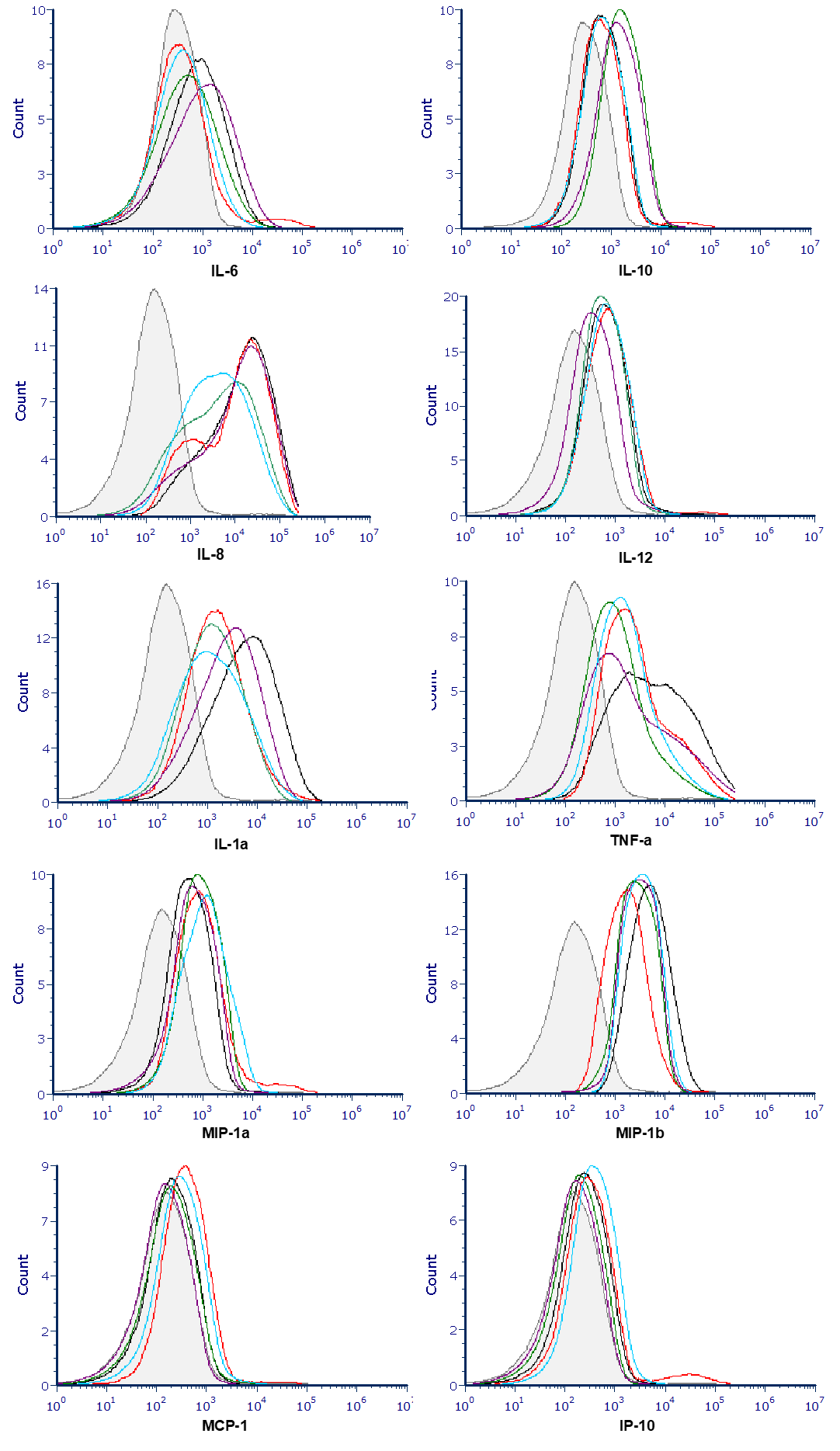


**Figure S1: Representative histograms of monocyte intracellular cytokine staining.** Histograms represent flow cytometry intracellular staining for monocytes gated as CD14^+^ in the model of bacterial infection (LPS). Plots include isotype control (grey filled), admission (black), OT (red), ICU (blue), D3 (green) and D5 (purple). As per manufacturer recommendations, a rat IgG isotype control was used for IL-6 and IL-10 staining. A mouse IgG isotype control was used for all remaining markers. All data normalised to sample with lowest event number (OT). X axis represents cytokine MFI. Y axis represents count.
